# Differences between the effects of conventional cigarettes, e-cigarettes and dual product use on urine cotinine levels

**DOI:** 10.18332/tid/100527

**Published:** 2019-02-18

**Authors:** Myung-Bae Park, Jung-Kyu Choi

**Affiliations:** 1 Department of Gerontal Health and Welfare, Pai Chai University, Dae Jeon, Republic of Korea; 2 Institute of Health Insurance and Clinical Research, National Health Insurance Corporation Ilsan Hospital, Goyang, Republic of Korea

**Keywords:** e-cigarette, cigarette, dual user, urine cotinine, South Korea

## Abstract

**INTRODUCTION:**

The goal of this study was to evaluate the differences in urine cotinine (UC) concentration based on the use of conventional cigarettes, e-cigarettes (ECs), and dual product use, and determine the use of ECs in the real world.

**METHODS:**

In total, 15099 subjects were classified into non-smokers, cigarette smokers (c-smokers), e-cigarette smokers (e-smokers), and dual users, and their UC (a classical biomarker of smoking) values were compared. Analysis of covariance (ANCOVA) was performed after adjusting for age, sex and job status to analyze the differences in UC concentration in relation to type of smoking. The reasons for using ECs and the experience of cigarette use before using ECs were analyzed.

**RESULTS:**

Of the 15099 people, 76.4% were non-smokers, 20.9% c-smokers, 2.3% dual users, and 0.4% were e-smokers. There were significant differences in UC concentration among the groups (p<0.001). The geometric mean (GM) UC concentration was 4.45 ng/mL. UC concentration was the highest among dual users (GM: 1030.5, median: 1258.9 ng/mL), followed by c-smokers (GM: 842.5, median: 1163.0 ng/mL), e-smokers (GM: 119.5, median: 309.7 ng/mL), and non-smokers (GM: 0.8, median: 0.8 ng/mL). Among the EC users, the rate of using ECs for health or social convenience was 81.9%. Among e-smokers, 11.4% had never smoked previously.

**CONCLUSIONS:**

The UC concentration was the highest among dual users. However, for the female population, the UC concentration was the highest among e-smokers. The vast majority of EC users were dual users. In addition, there were no differences in the frequency of cigarette smoking between the dual user and c-smoker groups. Consequently, EC use did not lead to a decrease in cigarette use, but did lead to an increase in UC concentration. Therefore, in the real world, dual users have higher cotinine levels than the other groups, which could indicate that they take more nicotine by cigarettes or ECs, or are more addicted than others.

## INTRODUCTION

There is an ongoing debate about electronic cigarettes (ECs). In some studies, the use of ECs reduced nicotine intake in smokers^1^, and ECs were reported to be less addictive than regular tobacco^2^. A review study of randomized controlled trials and cohort studies concluded that ECs were potentially helpful for the cessation of smoking and had few serious side effects^3,4^. Nevertheless, it has not been confirmed whether ECs are less harmful or beneficial to health. In another systematic review, EC use was found not to be associated with smoking cessation in the real-world^5^. Existing randomized controlled trials and cohort studies have used currently unpopular EC products or did not consider confounding variables in concluding that the evidence is unreliable and not definitive that smoking cessation is related to ECs^6^. In addition, the use of ECs has been reported to induce heart disease^7^ and have adverse effects on the respiratory, digestive, and neurological systems^8^.

Whether ECs should be recommended as a means to quit smoking, be prohibited or restricted remains controversial in academia and for government policies, across countries. Based on the conclusion that ECs are much less harmful than conventional cigarettes and are conducive to quitting smoking, the United Kingdom recommends ECs as part of nicotine replacement therapy (NRT)^9^. The Framework Convention on Tobacco Control (FCTC) of the World Health Organization (WHO) reached a unanimous consensus on regulating ads, promotion, and sponsorship of ECs in 2014, as their safety and efficacy cannot be definitively confirmed, and because they induce addiction similar to that caused by nicotine in cigarettes^10^. The US Food and Drug Administration (FDA) is striving to regulate ECs and has concluded that ECs are not recommended as a means to quit smoking for all smokers, including pregnant women, because there are many more aspects that require investigation, including their effects on smoking cessation^11^.

To address this debate, previous studies have stressed the need for further studies investigating the effects and harms of ECs^12^. Most studies to date have used non-representative samples^13^, which do not reflect the real world population. ECs are thought to be less harmful than cigarettes. However, there is the possibility that they will not be used in real life. Therefore, this study seeks to determine how ECs are used in real life through population-based surveys. To this end, we evaluated urine cotinine (UC) levels, which are a classical biomarker of nicotine uptake, among non-smokers, cigarette smokers (c-smokers), E-cigarette smokers (e-smokers), and dual users.

## METHODS

### Data and sample

We used data from the Korea National Health and Nutrition Examination Survey (KNHANEs). This survey investigated approximately 10000 people each year, and the stratified multistage sampling design (STRATA) was used to stratify the population by variables such as sex, age, and house type. Upon submission of simple personal information and research goals, these data can be downloaded with permission from the CDC KNHANE website (https://knhanes.cdc.go.kr/knhanes/eng/). The survey addressed EC use in people of age 19 years and over, from 2013 to 2016. UC data, however, was available from 2014. Therefore, among those who responded, 15099 participants with UC data between 2014–2016 were included in the study.

### Outcome measures

Current e-smokers were defined as those who answered ‘yes’ to the question: ‘Have you ever used an electronic cigarette?’. We also inquired about whether the use of an electronic cigarette had occurred within the past month. Individuals who smoked more than 100 cigarettes in their lifetime and people who currently smoked only conventional cigarettes were defined as current c-smokers, and those who fell under both definitions were defined as dual user smokers (dual user). In other words, e-smokers were people who smoked e-cigarettes for the past month but did not use c-cigarettes, c-smokers were people who smoked c-cigarettes but did not use e-cigarettes for the past month, and dual users were people who smoked e-cigarettes for the past month and used the c-cigarettes concurrently. Participant urinary cotinine concentration (UCC) was measured at the time of other KNHANEs investigations, in which an equipped mobile clinic visited target regions. The urine samples were collected to measure the UCC by random sampling. The UCC was tested annually in approximately 2000 people including children, but from 2016 all the subjects are investigated. UC was analyzed via high-performance liquid chromatography-tandem mass spectrometry (HPLCMS/MS) using the Agilent 1100 Series with API 4000 (AB Sciex, Framingham, MA, USA). Individuals who did not qualify as either an e-smoker, c-smoker, or dual user were defined as non-smokers. Because there is a high false response rate for smoking in East Asian countries^14^, non-smokers with a UC concentration of 100 ng/mL or greater were excluded from the analysis for a more accurate classification of current smokers^15^.

### Statistical analysis

Descriptive analysis was performed for age, sex, job status and form of cigarette smoking. Geometric means (GM) were used for analysis to account for the skewness of UC concentration^16^. Further, analysis of covariance (ANCOVA) analysis used log transformed values^17^, and it was performed after adjusting for age, sex and job status to analyze the differences in UC concentration in relation to type of smoking.

In addition, descriptive analysis was conducted for identifying the reason for using ECs in current e-smokers and dual users. Furthermore, descriptive analysis was performed on the experience of cigarette use among e-smokers. This was to identify whether e-smokers had become new smokers from previously being non-smokers. Weighted values were used for all analyses based on the stratified multistage sampling design.

## RESULTS

### Participant characteristics

Of the 15099 subjects, 76.4% were non-smokers, 20.9% were c-smokers, 2.3% were dual users, and 0.4% were e-smokers. Approximately 2.7% of the participants were current EC users. The mean participant age was 46.8 years. The mean age was the highest among non-smokers (48.0 years), followed by c-smokers (44.0 years), e-smokers (38.2 years), and dual users (35.5 years). The proportion of middle-aged participants (40–64 years) was the highest among c-smokers, but the percentage of young adults (19–39 years) was the highest among both e-smokers and dual users. The percentage of unemployed individuals, such as students and housewives, was the highest among non-smokers and dual users, but the percentage of white-collar workers was the highest among e-smokers and c-smokers. Only c-smokers and dual users answered the question about the number of cigarettes they smoked per day. The majority of participants smoked 10–19 cigarettes in both groups. The average number of cigarettes per day was 14.1 among c-smokers and 14.5 among dual users, as shown in Table 1 (see also Supplementary file, Table 1).

**Table 1:** Summary of study sociodemographics

	*Respondents (%)*	*Non-smokers (%)*	*E-smokers (%)*	*C-smokers (%)*	*Dual users (%)*
**Sex**	**15099 (100.0)**	**12182 (76.4)**	**44 (0.4)**	**2627 (20.9)**	**246 (2.3)**
Male	6606 (50.5)	4125 (29.8)	35 (0.3)	2229 (18.3)	217 (2.1)
Female	8493 (49.5)	8057 (46.6)	9 (0.1)	398 (2.6)	29 (0.2)
**Age group** (years)	**15099 (100.0)**	**12182 (76.4)**	**44 (0.4)**	**2627 (20.9)**	**246 (2.3)**
19–39	4046 (35.7)	3001 (25.4)	24 (0.2)	870 (8.5)	151 (1.6)
40–64	7167 (48.7)	5693 (36.9)	16 (0.1)	1374 (11.0)	85 (0.7)
≥65	3886 (15.6)	3488 (14.1)	4 (0.0)	384 (1.5)	10 (0.0)
**Mean age** (CL)	46.8 (46.4–47.3)	48.0 (47.5–48.5)	38.2 (34.2–42.2)	44.0 (43.3–44.6)	35.5 (34.0–37.0)
**Job status**	**14337 (100.0)**	**11651 (76.9)**	**43 (0.4)**	**2457 (20.5)**	**226 (1.2)**
White color	5070 (39.7)	3938 (29.3)	18 (0.2)	994 (9.0)	120 (0.6)
Blue color	3442 (23.7)	2526 (16.2)	11 (0.1)	847 (6.9)	58 (0.5)
Unemployed (include student, housewife)	5865 (36.6)	5187 (31.4)	14 (0.1)	616 (4.6)	48 (2.2)
**Cigarettes per day**	**15050 (100.0)**	**12182 (76.7)**	**44 (0.4)**	**2580 (20.6)**	**244 (2.3)**
non-cigarette smoker	12226 (77.1)	12182 (76.7)	44 (0.4)	-	-
1–9 (light smoker)	655 (5.0)	-	-	607 (4.6)	48 (0.4)
10–19 (moderate smoker)	1180 (9.9)	-	-	1066 (8.8)	114 (1.1)
≥20 (heavy smoker)	989 (8.0)	-	-	907 (7.3)	82 (0.8)
**Average cigarettes per day** (CL)	-	-	-	14.1 (13.8–14.5)	14.5 (13.6–15.5)

CL: 95% confidence limit for mean. All percentages are weighted to reflect stratified multistage sampling.

### Urine cotinine level of participants

The GM UC level of all subjects was 4.45 ng/mL. It was the highest among dual users (GM: 1030.5, median: 1258.9 ng/mL), followed by c-smokers (GM: 842.5, median: 1163.0 ng/mL), e-smokers (GM: 119.5, Median: 309.7 ng/mL), and non-smokers (GM: 0.8, Median: 0.8 ng/mL). In terms of sex, men showed a higher UC concentration in the c-smoker and dual user groups, but women showed a higher UC concentration (GM: 576.4, median: 937.8 ng/mL) than men (GM: 89.2, median: 217.0 ng/mL) in the e-smoker group. Occupation-wise, the GM was the highest among white collar workers in the e-smoker group (173.7 ng/mL) but the median value was similar for the white or blue collar workers. Meanwhile, e-smokers showed a greater range of UC concentration compared to that of the other two groups. In the c-smoker group, blue collar workers had the highest UC concentration. In the dual user group, blue collar workers had the highest GM (1314.7 ng/mL), but white collar workers showed a slightly higher median value. In terms of form of smoking, heavy smokers showed the highest UC concentration in both the c-smoker and dual user groups (Table 2).

**Table 2:** Geometric mean and median of level of urine cotinine in the non-, electronic-, and conventional smokers, and dual users (ng/mL)

	*n*	*Non-smokers*		*E-smokers*		*C-smokers*		*Dual users*	
		*Median (IQR)*	*GM (CL)*	*Median (IQR)*	*GM (CL)*	*Median (IQR)*	*GM (CL)*	*Median (IQR)*	*GM (CL)*
		0.8 (0.4–1.8)	0.8 (0.2–0.8)	309.7 (4.9–1264.4)	119.5 (53.9–49.2)	1163.0 (618.0–1697.9)	842.5 (792.2–896.0)	1258.9 (757.0–1836.8)	1030.5 (910.9–1165.7)
**Sex**	**15099**								
Male	6606	1.6 (0.5–0.8)	0.9 (0.9–1.0)	217.0 (4.2–1001.0)	89.2 (32.4–245.7)	1230.0 (686.0–1765.7)	903.3 (846.4–964.1)	1310.0 (843.8–1900.2)	1116.9 (991.2–1258.5)
Female	8493	1.5 (0.4–1.5)	0.8 (0.8–0.8)	937.8 (115.0–1700.0)	576.4 (231.2–1437.0)	800.0 (414.2–1300.0)	517.9 (431.3–622.0)	717.8 (1154–230.3)	426.0 (241.6–751.2)
**Age group (years)**	**15099**								
19–39	4046	1.5 (0.4–0.7)	0.9 (0.8–0.9)	581.0 (64.4–1264.4)	189.0 (75.5–473.3)	1.105.0 (488.0–1680.0)	756.1 (679.8–841.0)	1119.2 (680.1–1778.3)	915.5 (773.0–1084.2)
40–64	7167	0.8 (0.4–1.6)	0.9 (0.8–0.9)	419.1 (3.0–1580.0)	61.8 (7.9–480.1)	1263.6 (739.0–1807.2)	935.9 (868.2–1008.9)	1450.9 (1010.0–1987.2)	1368.5 (1208.4–1549.8)
≥65	3886	0.7 (0.4–1.4)	0.8 (0.7–0.8)	3.8 (1.7–30.4)	4.7 (0.5–44.4)	868.2 (497.5–1360.0)	719.6 (647.5–799.7)	551.0 (235.0–1175.3)	659.3 (296.5–1466.2)
**Job status**	**14337**								
White color	5070	0.8 (0.4–1.6)	0.9 (0.8–0.9)	456.2 (110.5–986.8)	173.7 (62.0–486.4)	1124.8 (530.0–1640.0)	721.8 (547.4–804.7)	1320.9 (816.7–1830.2)	1020.8 (860.9–1210.3)
Blue color	3442	0.9 (0.5–1.7)	0.9 (0.9–1.0)	459.7 (1.2–1700.0)	99.3 (15.4–640.8)	1330.6 (808.1–1890.0)	1062.2 (983.5–1147.0)	1273.2 (951.0–1967.7)	1314.7 (1068.2–1618.0)
**Unemployed** (include student, housewife)	5865	0.7 (0.4–1.4)	0.8 (0.7–0.8)	34.6 (3.3–1544.7)	61.1 (7.1–529.6)	917.3 (545.0–1449.6)	737.8 (645.1–843.9)	952.0 (512.3–1520.6)	839.3 (593.9–1186.2)
**Cigarettes per day**	**15050**								
non-cigarette smoker	12226	0.8 (0.4–1.5)	0.8 (0.8–0.9)	309.7 (4.9–1264.4)	119.5 (49.2–290.0)	-	-	-	-
1–9 (light smoker)	655	-	-	-	-	546.0 (210.6–972.0)	341.6 (294.5–396.2)	693.9 (262.1–1063.7)	492.9 (350.0–694.1)
10–19 (moderate smoker)	1180	-	-	-	-	1178.4 (718.0–1650.0)	991.4 (926.9–1060.5)	1252.0 (843.8–1693.8)	1049.7 (910.4–1210.3)
≥20 (heavy smoker)	989	-	-	-	-	1538.0 (1120.0–2080.7)	1435.3 (1360.5–1514.2)	1785.8 (1181.8–2459.3)	1567.0 (1318.3–1862.7)
**Average cigarettes per day** (CL)	-	-	-	-	-	14.1 (13.8–14.5)	-	14.5 (13.6–15.5)	-

IQR: interquartile range, GM: geometric mean, CL: 95% confidence limit for mean.

**Figure 1:**
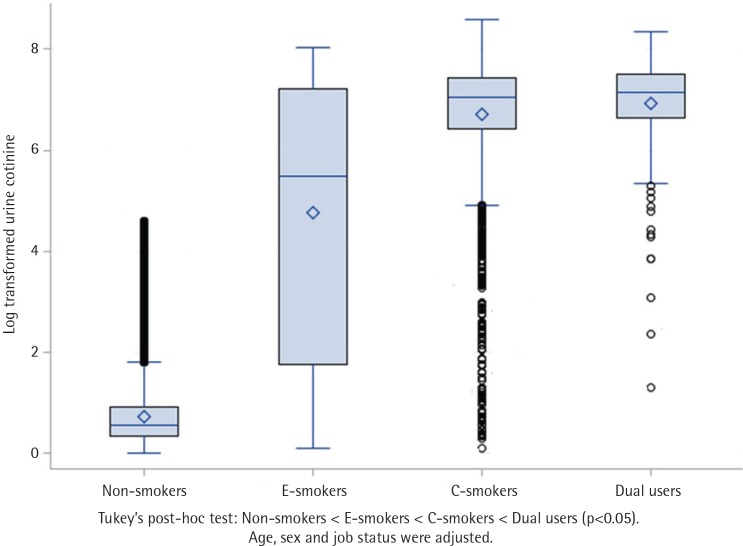
Difference in urine cotinine level according to ANCOVA analysis

**Figure 2:**
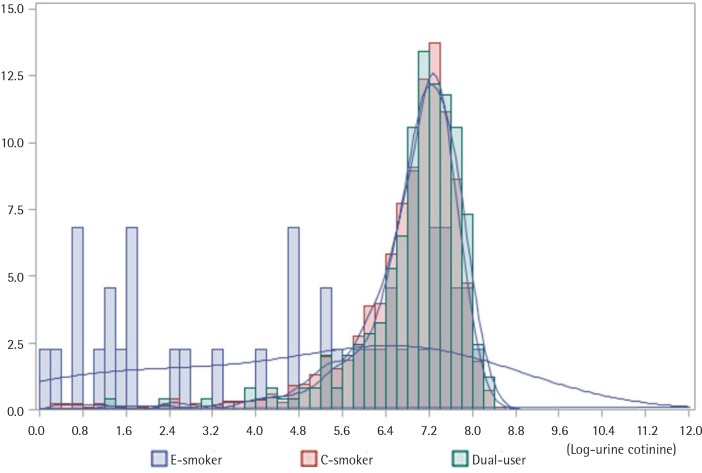
Urine cotinine concentration in E-smokers, C-smokers, and Dual users

Additionally, we performed an analysis of covariance (ANCOVA) to confirm whether these differences in UC concentration were statistically significant. There were significant differences in UC concentration among the groups (p<0.001). Tukey’s post-hoc test revealed that the dual user group had the highest UC concentration, followed by the c-smoker, e-smoker, and non-smoker group (Figure 1). In addition, to confirm the distribution of UC concentration, a histogram was used. At high UC concentration, the degree of dual-usage was relatively high (Figure 2).

### Reasons for EC use and cigarettes smoking history

We analyzed the reasons for EC use in ECs users. For the question: ‘What are the main reasons for using electronic cigarettes?’, only one major reason provided was recorded. The results of responses were as follows: ‘To try and quit smoking’ (45.2%), followed by ‘Less harmful than regular cigarettes’ (19.4%), ‘Curiosity’ (15.6%), ‘Reduced odor compared to regular cigarettes’ (12.8%), and ‘It is easy to smoke indoors’ (4.5%) (Table 3). In addition, regarding prior experience of cigarette use among e-smokers, 88.6% of 44 responders had prior experience, while 11.4% did not have prior experience (Table 4).

**Table 3:** Reason for e-cigarette use

	Respondents (weighted frequency*) N= 225 ( 2179142 )	*Weighted percentage*
Less harmful than regular cigarette	**43 (422095)**	**19.4**
To try to quit smoking	**101 (985444)**	**45.2**
It is easy to smoke indoors	**10 (97187)**	**4.5**
Easier to get than regular cigarette	0 (0)	0.0
Taste good	2 (15547)	0.7
Less smell than regular cigarette	**28 (279867)**	**12.8**
Curiosity	36 (339551)	15.6
Etc.	4 (32309)	1.5
Don’t know	1 (7141)	0.3

* Estimated number of users based on sample weight.

**Table 4:** Conventional cigarettes smoking history before using electronic cigarettes

	*Respondents (weighted frequency †) N= 44 ( 400939 )*	*Weighted percentage*
People with a conventional cigarette using experience*	39 (365617)	88.6
Never smoker**	5 (35322)	11.4

† Estimated number of users based on sample weight.

* People with a conventional cigarette using experience before using e-cigarette; it is unclear whether they are conventional cigarette smoker or not, just before the start of e-cigarette use.

** Never smoker (non-smoker in their lifetime); 11.4% non-smokers became e-smokers.

## DISCUSSION

In the present study, e-smokers had a significantly lower UC concentration compared to c-smokers and dual users. However, dual users had a higher UC concentration than that of the c-smokers, and there were no differences in the amount of smoking between the dual user and c-smoker groups. A previous review concluded that e-smokers and c-smokers do not differ in their nicotine or cotinine concentrations^18^, while Göney et al.^19^ reported in their study on 100 participants that there was no difference in UC concentration between EC users and c-smokers. In the Hecht et al. review^20^ concentrations of toxicant and carcinogen metabolites, including cotinine, were lower in the EC group compared to that in the c-smoker group; however, they had a small sample size, which included individuals who were attempting to quit smoking using ECs; the study also did not consider dual users. McRobbie et al.^21^ on 40 individuals desiring to quit smoking reported that individuals showed a reduction of nicotine and acrolein concentration after converting to e-smoking or dual-usage. In the Polosa et al.^22,23^ and Campagna et al.^24^ cohort studies, ECs were reported to be helpful for reducing the amount of smoking or quitting smoking; however, the study had a small sample size, aimed to help participants quit smoking, and was funded by EC companies. In summary, most studies on ECs have drawn their conclusions based on a non-realistic environment, small sample size, or artificial interventions attempting to help quit smoking. In this context, Kalkhoran and Glantz^5^ concluded that ECs would have an unlikely association with smoking cessation in the previous studies were they in the real-world and not in a clinical setting.

In terms of UC concentration according to smoking type, UC was the highest in dual users in general, while cigarette use was similar to that of c-smokers. In other words, the UC concentration was the highest among dual users regardless of age, occupation, and number of cigarettes per day. Due to the cross-sectional design of this study, we could not assess whether using ECs is helpful in quitting smoking or not. Therefore, it is true that dual users had the highest nicotine levels, but we cannot infer whether dual users are more likely to quit smoking in the future. In this study, c-smokers and dual users used almost the same numbers of cigarettes per day, which suggests that dual users do not reduce the number of cigarettes smoked, and rather add e-cigarette use. Even if e-cigarettes have the mid- to long-term effect of quitting smoking, there is a need to prioritize interventions for dual users in the real world because UCC is higher in this group than any other group. In this study, certain trends are observed based on sex. In general, men have higher levels of cotinine because they smoke more cigarettes than women; however, in this study, UC concentration was higher among female smokers than among male smokers^25^. EC advertisements focusing on the younger generation are prevalent. Because of this, the risks associated with EC use or proper use after consultation with a relevant professional for smoking cessation, needs to be made clear to younger age groups.

In order to confirm whether ECs are less harmful to a person’s health, the assessment should be restricted to the exclusive use of ECs by c-smokers in order to quit smoking or reduce cigarette consumption. In other words, c-smokers who are trying to quit smoking should be prevented from becoming dual users or the period of dual-usage should be reduced.

Previous studies have reported that the major reason for using ECs is to quit smoking, because they are less harmful^26^, due to social stigma, or for convenience^27^. In the present study, 81.9% of the population was using ECs (e-smokers, dual users) for the same reasons mentioned above (Table 3). In other words, a substantial number of EC users in the real world had begun using e-cigarettes for health promotion and social enhancement, but most of them actually remained as dual users and thus of poorer state. Considering this situation, ECs may be effective for quitting smoking in limited situations. As shown in our findings, the group of people using only ECs had a relatively lower nicotine dependence compared to other smoker groups. However, in the real world, the percentage of people using only ECs is low, and most become dual users, and so are of poorer state. Chapman^28^ also reports that becoming a dual user is a threat to quitting smoking. In addition, although it is true that the UC concentration of people who use only ECs is relatively low, the standard deviation of smoking amount is so large that e-smokers with high UC concentrations (heavy e-smoker) should be properly monitored and perhaps targeted interventions specific to e-smokers are needed. According to the most recent research (2018), ECs should be well designed and implemented to help quit smoking^18^. In order to use ECs as NRTs for smoking cessation in the future, in countries such as Korea that do not use ECs as aids to quit smoking, the adverse effects of increased proportions of dual users is likely to occur without appropriate policy interventions such as expert guidance on ECs. Hence, adverse effects of ECs on health promotion or smoking cessation should be addressed in the form of new health policies.

The question remains as to whether or not ECs are helpful for quitting smoking. The EC market has grown rapidly amid this debate^29^ and EC companies aggressively promote their products as a means to improve health. In addition, EC market techniques are evolving, such as the development of various flavors^30^. EC marketing by transnational tobacco companies (TTC) has become the greatest threat to tobacco control^12^. Once exposed to EC ads, people are highly likely to use them^31^; however, instead of being effective in promoting smoking cessation, non-smokers become e-smokers or dual users and are exposed to more adverse effects on health^32^. Sussan et al.^33^ reported that the proportion of former smokers among EC users is high. This means that a person who quits tobacco is re-smoking through ECs. Furthermore, approximately 6% of never smokers became e-smokers. In this study, 11.4% of e-smokers were never smokers and became new smokers through ECs. Therefore, if ECs are not to be used as NRTs, it is important to implement appropriate regulations against ECs^32,34^.

Timely assessments should be made to determine whether ECs should be recommended medically and whether health policies to regulate ECs are needed. Until this policy is developed, governments that recommend the use of ECs should intervene to guide and educated people who chose to use them as an aid to quit smoking. Further, the vast majority of countries that do not acknowledge ECs as an aid to quit smoking should implement multidimensional efforts, such as strong legal regulations, campaigns, and health education, to prevent new e-smokers or dual users. Additionally, because ECs themselves are a health hazard^7,8,35^, governments should caution against the conversion of a non-smoking status into new EC status due to the aggressive marketing by ECs companies.

### Limitations and strengths

This study is one of the few studies to determine the smoking status of e-smokers, c-smokers, and dual users through assessment of biomarkers. It is also meaningful because we have confirmed that the use of ECs may be accompanied by adverse effects in the form of new e-smokers or dual users. However, this is a cross-sectional study that cannot explain the effect of ECs on smoking cessation, and there is the limitation that the UCC alone is not sufficient to assess health risks.

## CONCLUSIONS

Those who used only ECs (e-smokers) had a lower UC concentration than conventional cigarette users (c-smokers). However, UC concentration among dual users was higher than that of c-smokers. The deviation of UC concentration is so large among e-smokers, thus heavy e-smokers should be properly controlled. ECs users are far more likely to be dual users than exclusive e-smokers; although this study did not confirm the exact number, a number of never smokers became e-smokers. This study confirmed the adverse effects of using ECs. Therefore, it is necessary to strengthen policies, such as stricter regulations of ECs, or more appropriate interventions for smoking cessation in the real world. Finally, special consideration for women is needed when prioritizing policies for ECs, since they have a high nicotine dependency resulting from EC use.

## CONFLICTS OF INTEREST

Authors have completed and submitted the ICMJE Form for Disclosure of Potential Conflicts of Interest and none was reported.
