# Supplementary material for: Differences between the effects of conventional cigarettes, e-cigarettes and dual product use on urine cotinine levels
Source: Tob Induc Dis. 2019 Feb 18;17:12. doi: 10.18332/tid/100527 (PMC6751982; doi:10.18332/tid/100527)
Supplement: Supplementary file 1 [file TID-17-12-s1.pdf]

Supplementary Table 1. Summary of study sociodemographics (weighted frequency reflected the population in South Korea)

	Respondents (%)	Non-smokers (%)	E-smokers (%)	C-smokers (%)	Dual users (%)
**Sex**	**106084073(100.0)**	**81018283(76.4)**	**400939(0.4)**	**22221952(20.9)**	**2442899(2.3)**
Male	53625455(50.5)	31612511(29.8)	338254(0.3)	19435845(18.3)	2238846(2.1)
Female	52458618(49.5)	49405772(46.6)	62685(0.1)	2786107(2.6)	204053(0.2)
**Age group** (years)	**106084073(100.0)**	**81018283(76.4)**	**400939(0.4)**	**22221952(20.9)**	**2442899(2.3)**
19–39	37870467(35.7)	26952023(25.4)	264100(0.2)	9004824(8.5)	1649519(1.6)
40–64	51662448(48.7)	39150629(36.9)	124811(0.1)	11634592(11.0)	752417(0.7)
≥65	16551158(15.6)	14915631(14.1)	12028(0.0)	1582536(1.5)	40963(0.0)
**Mean age (CL)**	46.8(46.4–47.3)	48.0(47.5–48.5)	38.2(34.2–42.2)	44.0(43.3–44.6)	35.5(34.0–37.0)
**Job status**	**100747676(100.0)**	**77428498(76.9)**	**387354(0.4)**	**20685899(20.5)**	**2245925(1.2)**
White color	40004647(39.7)	29516505(29.3)	181582(0.2)	9111675(9.0)	1194885(0.6)
Blue color	23894252(23.7)	16278304(16.2)	80027(0.1)	6959686(6.9)	576234(0.5)
**Unemployed** (include student, housewife)	36848778(36.6)	31633689(31.4)	125745(0.1)	4614538(4.6)	474806(2.2)
**Cigarettes per day**	**105631975(100.0)**	**81018283(76.7)**	**400939(0.4)**	**21804270(20.6)**	**2408483(2.3)**
non-cigarette smoker	81419222(77.1)	81018283(76.7)	400939(0.4)	-	-
1–9 (light smoker)	5310975(5.0)	-	-	4853121(4.6)	457854(0.4)
10–19 (moderate smoker)	10407564(9.9)	-	-	9291406(8.8)	1116158(1.1)
≥20 (heavy smoker)	8494214(8.0)	-	-	7659743(7.3)	834472(0.8)
**Average cigarettes per day** (CL)	-	-	-	14.1(13.8–14.5)	14.5(13.6–15.5)

CL: 95% confidence limit for mean.

© 2019 Park M. B.
